# A survey of factors affecting clinician acceptance of clinical decision support

**DOI:** 10.1186/1472-6947-6-6

**Published:** 2006-02-01

**Authors:** Dean F Sittig, Michael A Krall, Richard H Dykstra, Allen Russell, Homer L Chin

**Affiliations:** 1 Department of Medical Informatics, Northwest Permanente PC, Portland, OR, USA; 2 Department of Medical Informatics and Clinical Epidemiology, Oregon Health & Science University, Portland, OR, USA; 3 Center for Health Research, Kaiser Permanente, Northwest Portland, OR, USA

## Abstract

**Background:**

Real-time clinical decision support (CDS) integrated into clinicians' workflow has the potential to profoundly affect the cost, quality, and safety of health care delivery. Recent reports have identified a surprisingly low acceptance rate for different types of CDS. We hypothesized that factors affecting CDS system acceptance could be categorized as relating to differences in patients, physicians, CDS-type, or environmental characteristics.

**Methods:**

We conducted a survey of all adult primary care physicians (PCPs, n = 225) within our group model Health Maintenance Organization (HMO) to identify factors that affect their acceptance of CDS. We defined clinical decision support broadly as "clinical information" that is either provided to you or accessible by you, from the clinical workstation (e.g., enhanced flow sheet displays, health maintenance reminders, alternative medication suggestions, order sets, alerts, and access to any internet-based information resources).

**Results:**

110 surveys were returned (49%). There were no differences in the age, gender, or years of service between those who returned the survey and the entire adult PCP population. Overall, clinicians stated that the CDS provided "helps them take better care of their patients" (3.6 on scale of 1:Never – 5:Always), "is worth the time it takes" (3.5), and "reminds them of something they've forgotten" (3.2). There was no difference in the perceived acceptance rate of alerts based on their type (i.e., cost, safety, health maintenance). When asked about specific patient characteristics that would make the clinicians "more", "equally" or "less" likely to accept alerts: 41% stated that they were more (8% stated "less") likely to accept alerts on elderly patients (> 65 yrs); 38% were more (14% stated less) likely to accept alerts on patients with more than 5 current medications; and 38% were more (20% stated less) likely to accept alerts on patients with more than 5 chronic clinical conditions. Interestingly, 80% said they were less likely to accept alerts when they were behind schedule and 84% of clinicians admitted to being at least 20 minutes behind schedule "some", "most", or "all of the time".

**Conclusion:**

Even though a majority of our clinical decision support suggestions are not explicitly followed, clinicians feel they are of benefit and would be even more beneficial if they had more time available to address them.

## Background

In response in part to the Institute of Medicine's reports "Crossing the Quality Chasm" [1] and "To Err is Human" [2], and the American Medical Informatics Association's position paper on the use of clinical decision support in electronic prescribing [3] there is increased pressure to implement state of the art clinical information systems (CISs) with real-time clinical decision support capabilities. Unfortunately, several recent reports have documented that a disturbingly high percentage (i.e., 54 – 91%) of real-time clinical decision support suggestions are being over-ridden, or ignored, by clinicians [4-6].

Granted, there are certainly cases in which "overriding" the computer-generated alert is the correct action on the part of the clinician including: the benefits of the action outweigh the risks, there is no good alternative, this is an "expected" side-effect of a particular therapy or procedure, or that the medication was previously or currently tolerated, to name just a few. On the other hand in all of the studies cited above, in which the clinicians overrode a very high percentage of all alerts, the authors found that in almost all cases the computer-generated alerts were "true-positives" meaning that most observers would consider the clinicians actions to ignore the alert to be contrary to "best clinical practices".

We are in the process of designing, implementing, and evaluating many new clinical decision support features and interventions [7,8]. Based on our knowledge of the literature and extensive clinical informatics experience, we recognize that there are a myriad of factors associated with clinicians' refusal to accept, or follow, computer-generated, care suggestions based on clinical guidelines including lack of: awareness that the guideline even exists, familiarity with the recommendation, agreement with the suggestion, belief that they could even perform the expected behavior (often referred to as: self-efficacy), belief that the expected improvement in outcome will occur, ability to overcome the inertia of previous practice, and the existence of external barriers to the performance of the recommendations (e.g., no time or no reminder system) [9]. In addition to these mostly internal, provider-related factors, there are also many computer-related hypotheses for why clinicians refuse to follow these suggestions including: failure to provide patient-specific information (which was not shown to be a factor in this study) [10], specific aspects of the human-computer interaction surrounding the presentation of the reminders, for example, presenting fully-completed orders that follow the guideline on the same screen as the reminder, rather than placing them "one click away", using a distinctive color scheme to "highlight" the recommendation, disabling the escape key which made it more difficult to override the suggestion, setting the default value of the suggestion to "order" rather than "not to order", and presenting the same reminder over and over to all clinicians who viewed a particular patient's data (i.e., until the suggestion was accepted) [11]. While we were not able to follow all of these "best practices" for the design of interactive clinical decision support features due to inherent limitations of our commercially available EMR and some institutional resistance on the part of clinical and information system administrators, we are doing our best to remove as many potential barriers as possible.

We hypothesized that there are other factors that may account for clinicians' refusal to follow computer-generated clinical suggestions with the intended action of removing, or at least reducing, as many of the identified barriers as we can. Therefore, we undertook this study to begin exploring these other potential factors affecting clinician acceptance of clinical decision support at the point of care.

### Clinical computing environment

We conducted the survey within Northwest Permanente, the physicians' group associated with Kaiser Permanente, Northwest (KPNW) in Portland, OR. Briefly, KPNW is a large, group model health maintenance organization serving northwestern Oregon and southwestern Washington. KPNW is a pre-paid medical plan that is responsible for the health of over 455,000 patients. KPNW implemented a commercially available ambulatory medical record product from Epic Systems (Madison, WI) beginning in 1994 and was fully implemented in 1997. In 1998, they won the Nicholas E. Davies Award for CIS implementations [12]. In 2003 and again in 2005, KNPW was voted the best HMO by survey respondents of a leading consumer magazine [13,14].

### Clinical decision support within KPNW

Over the past several years, a number of careful assessments of the effects of various clinical decision support features have been made using the clinical information system within KPNW including: 1) using an off-line data analysis technique to identify patients eligible for a specific alert that could be presented to the clinician [15]; 2) the effect of alerts that remind clinicians about medications contraindicated in the elderly [16]; 3) the effect of alerts that recommend dose changes in patients with various levels of renal insufficiency [17]; and 4) the effect of alerts that notify clinicians in the event that a patient is on, or being prescribed two medications that may have a serious interaction [18]. All of these studies showed significant and sustained benefits to patients. That is, the percentage of patients receiving the contraindicated medications decreased by approximately 10–20% relative to the baseline measurements after 12 months of continuous usage. While this decrease was statistically, as well as, clinically significant, there were still patients who continued to receive these contraindicated medications which means that the clinicians, ignored or overrode many of the alerts. These findings led us to begin asking clinicians questions about the clinical decision support that we were providing.

### The survey

Based on the work of several investigators [19,20] we hypothesized that clinicians' acceptance of clinical decision support could be explained by one, or a combination of, factors from the following categories:

■ **Patient**: reason for visit, severity of illness – estimated based on the number of medications the patient was taking and the number of chronic conditions they had, or age.

■ **Provider**: age, gender, or number of years with Kaiser Permanente.

■ **Alert**: type of alert or number of alerts received.

■ **Environment**: examination room set-up including presence of a computer or estimated number of minutes the clinician is behind schedule.

We defined clinical decision support as "clinical information that is either provided to you or accessible by you, from the EpicCare clinical workstation". We consider enhanced information displays such as flow sheets, health maintenance reminders, alternative medication suggestions, order sets or smart sets, alerts, and access to any internet-based information resources like the KPNW Clinical Library as clinical decision support.

## Methods

Following Institutional Review Board (IRB) approval, we sent an anonymous, 2-page survey (see Additional file 1) along with a cover letter that introduced the project, to all 225 adult primary care physicians (i.e., all physician members of the Internal Medicine and Family Medicine departments) via interdepartmental mail. Completed surveys were returned over a 5-week period. No follow-up attempts were made to increase the survey return rate out of respect for clinicians' valuable time and attention and fear of compromising our ability to use these same clinicians as subjects in future research studies.

The questions were designed to identify potential factors affecting CDS system acceptance as well as specific system usage patterns on the part of clinicians. In addition, we asked a few questions to help us identify potential differences in CDS system acceptance that might be explained by basic demographic characteristics. Most of the questions could be answered using a 5-point Likert scale where 1 represented "Never"; 2 = "Rarely"; 3 = "Some of the time"; 4 = "Most of the time"; 5 = "Always". A few questions asked clinicians to answer questions based on whether they were "More", "Less", or "Equally" likely to behave in a certain way. Finally, we asked a few "open-ended" questions to allow respondents to answer in their own words. These responses were then coded for further analysis.

Following manual entry of the survey results, we calculated the mean, standard deviation, and range of responses for each numeric answer. For each open-ended, text-based answer field, we coded and tabulated the results according to frequency of occurrence. For several key variables we calculated cross tabulations in an attempt to identify interesting correlations between variables.

## Results

### Provider characteristics

Table 1 compares several demographic characteristics of the physicians who returned the survey to those of all physicians in the medical group. There were no significant differences in any of the characteristics we measured between those providers who returned the survey and those who did not.

**Table 1:** Demographic characteristics of survey respondents compared to the entire group of adult primary care physicians at Kaiser Permanente, Northwest.

	**Respondents**	**Entire Medical Group**
Number of individuals	110	225
Gender distribution	62% male	62% male
Mean age	46.5 years	46.1 years
Tenure at Kaiser Permanente	11.7 years	10.5 years

#### Differences in responses by gender

On average, female providers were younger (f: 43.1 vs. m: 48.7 yrs.) than their male counterparts and had been at KPNW less time (f: 7.9 vs. m: 14.2 yrs.), were more likely to accept "safety-related" alerts (f: 3.76 vs. m: 3.39), more often "relieved" to get an alert (f: 2.57 vs. m: 2.16), more often felt "empowered" when receiving an alert (f: 2.66 vs. m: 2.32), more likely to enter their notes into the computer in the examination room (f: 2.52 vs. m: 2.25), and more likely to admit that they were more than 40 (f: 2.65 vs. m: 2.44) or 60 (f: 1.96 vs. m: 1.78) minutes behind schedule. None of the other responses differed between the sexes.

#### Differences in responses based on years with Kaiser Permanente

Respondents, who had been with KPNW for 1–3 years, were more likely to report feeling relieved (1: 66% vs. 15: 30%), feeling grateful (1: 91% vs. 15: 69%), to "show the patient's their information" (1: 100% vs. 15: 66%) and to admit that "it reminded them" of something they had forgotten (1: 91% vs. 15: 69%), "some", "most" or "all" of the time, than those who had been with KPNW for more than 15 years. There were no other significant differences in the responses based on the number of years the clinicians had been with Kaiser Permanente.

### Overall response to clinical decision support

When asked to "rate" the clinical decision support that is currently being offered within the clinical information system, respondents were fairly positive, reporting that "It helps me take better care of my patients." (3.5), "It's worth the time it takes." (3.5), and "It reminds me of something I had forgotten about." (3.1).

When asked about their emotional responses when viewing alerts that occur during the medication order entry process, clinicians were on average more positive (mean for all positive emotions = 2.8; range 3.3 – 2.3) than negative (mean = 2.1; Range 2.4 – 1.8). The only other emotion reported more than once on the "fill-in-the-blank" portion of the question was "Annoyed".

### Alert/Reminder characteristics

There was no difference in clinicians' response to the question of how often they accept each of the three different types of clinical decision support alerts (cost-related: 3.6; safety-related: 3.5; health maintenance: 3.4). In addition, we found no differences in reported alert type acceptance based on gender, number of minutes they were behind schedule, or years with Kaiser Permanente.

### Environmental factors

When asked about specific uses of the clinical information system in the examination room, respondents reported that they were most likely to use it to "Look up patient information." (3.9), "Enter orders for the patient." (3.8), "Show the patient a graph of his/her laboratory values, wt, blood pressure, or growth" (2.9), "Use KPNW Clinical Library or other reference information" (2.4), and "Enter their progress/visit note" (2.4).

When asked how often they were behind schedule, more than 84% of clinicians reported that they were "more than 20 minutes behind schedule" some, most, or all of the time. In addition, women physicians were 40% more likely than their male counterparts to report that they were greater than 60 minutes behind schedule some or most of the time.

Interestingly, when we broke the responses down by how often respondents reported being behind schedule, we found that those who were the most behind were less likely to have access to computers in their examination rooms (less behind: 19% vs. most behind 36%; i.e., reported that they "never" or "rarely" had access to computers in their exam rooms). There were no differences in the reported likelihood that either those who were behind a lot or a little regarding their acceptance of any of the alert types.

### Patient-related factors

Table 2 presents the results of the questions that addressed specific patient characteristics that might make them MORE, LESS, or EQUALLY likely to accept specific health maintenance reminders.

**Table 2:** Patient characteristics that might influence clinician acceptance of alerts.

	**MORE**	**LESS**	**EQUALLY**
**Elderly patients (> 65 years old)**	40%	11%	49%
**Many current medications (> 5)**	36%	15%	49%
**Many chronic conditions (>5)**	36%	20%	44%
**Presenting with Acute Problem**	9%	59%	32%

#### Responses to open-ended survey questions

Respondents stated that they looked up information on medications "some of the time" during the patient visit (3.0). Their favorite information resource which was written in on 62 of the 110 responses was Micromedex Drug Information (e.g., DrugPoints from Thomson Micromedex Healthcare Series, 2005). The only other information resources that were mentioned more than 5 times were Epocrates (8) and the Physician's Desk Reference (PDR) (8).

Overall, clinicians stated that if allowed, they would decrease (61%) the number of alerts (which they estimated at 7.6 alerts/0.5 day shift) they were receiving. Finally, when asked "if you could turn off one alert?" their most common response was from the category of "Health Maintenance Reminders" (e.g., Aspirin, Pneumovax, HbA1c, "reminder to chart the patient's smoking status). Other common suggestions for alerts to turn off included:

1) "Drug-drug interactions" (e.g., several medications that interacted with Warfarin were specifically mentioned), which they did not like since many of the patients' medication lists were not up to date thus giving false positive alerts;

2) "Drugs to be avoided in the elderly" especially muscle relaxants; and

3) Several alerts that come up at the wrong point in the work flow, for example, alerts that display whenever a clinician opens a patient's chart or those that appear when the clinician is charting a "telephone encounter" since in these cases the patient is not even in the presence of the clinician.

## Discussion

Perhaps the most interesting finding was that specific patient characteristics were associated with the decision to accept or ignore various clinical decision support features. Specifically, clinicians indicated they were more willing to accept clinical decision support when the patient was elderly, had multiple medications or chronic conditions, and much less likely to accept the computer suggestions when the patient was presenting for an acute condition. Since all of these patient characteristics are known prior to the visit, it is possible that the organization could eliminate the presentation of the alerts in the urgent care setting. However while possible, there is uncertainty as to whether such an action would be appropriate. For example, many clinician leaders feel that every opportunity should be taken to provide preventive procedures or health maintenance screening tests to our membership, including when patients present with acute conditions. On the other hand, some front line clinicians feel that it is not their responsibility to address these deferrable events during visits in the urgent care clinic.

The other interesting finding was how often clinicians reported that they were behind schedule and that these clinicians were much less likely to accept alert suggestions when they were behind schedule. This raises the difficult question of whether we should "turn off" some or all of the alert types when the clinicians are significantly behind schedule. While one could argue that this would save the clinicians a little time, one could also argue that during these times of extraordinary stress is exactly when the alerts are the most needed.

It was difficult, if not impossible, for us to separate the differences in responses due to gender from that of the number of years with Kaiser Permanente since those clinicians who have been with Kaiser Permanente for 1–3 years are predominately female (73%) while those with more than 15 years of service are 90% male. Therefore, we caution readers not to make too much of the fact that either gender or years of service with Kaiser Permanente has significant affects on clinicians acceptance of various clinical decision support features since both of these factors clearly have significant affects on many aspects of one's life.

The decision of whether to include estimates of the amount of time a clinician is behind schedule with the provider or environmental factors may be controversial since clinicians within our organization do not have any management authority over their office or medical assistant staffing. Therefore, clinicians have little control over how their day is structured and may get behind schedule due to many different reasons including a) his/her particular practice style that does not mesh well with the 20-minute visit schedule and the unpredictable nature of clinical encounters coupled with the increasing demands and expectations for care and service during these encounters, b) inefficiencies on the part of the clinic staff in preparing the examination rooms and bringing the patients in from the waiting room and taking their vital signs, etc., c) patients often arrive late for their scheduled visit and are "worked" into the remaining schedule for the day, or d) and perhaps more likely, a combination of one or more of these and other less well identified factors.

Regardless of the appropriate categorization of this factor, or why the clinicians are behind schedule, there was a strong correlation between those physicians who reported being behind schedule and several key practices that our organization is trying to promote, including accepting safety alerts, looking up information on specific medications, and using on-line reference information. Of specific interest to our organization's leadership was the fact that the clinicians who reported being behind schedule more often also reported that they had less access to computers in their examination rooms. This information coupled with another recent report on computers in the exam room that associated improvements in patient satisfaction in three distinct areas a) satisfaction with visit components; (b) comprehension of the visit; and (c) perceptions of the physician's use of the computer [21] has helped justify a recent decision to equip all of our examination rooms with computers.

As a result of this study and several other co-occurring efforts, the clinical and administrative leadership of Kaiser Permanente, Northwest, temporarily turned off all health maintenance reminders and re-examined all of their logic and trigger points. After several weeks the new, and improved, alerts were re-initiated region-wide.

### Study limitations

The main limitation of this study was the small sample size of the survey and the fact that all respondents were members of the same HMO and utilized the same electronic medical record-keeping system. While there were no easily identifiable differences between the respondents and the rest of the clinician group, this does not mean that there was not some other, and possibly very important, unmeasured difference. In addition, the HMO setting of the study may overestimate the positive responses to the clinical decision support on the part of clinicians since some of the reminders were for factors that played a small and indirect role in the clinicians' year-end financial compensation, as a component of a system wide quality performance measure. Whether clinical decision support should be, or can be, used to help clinicians reach specific clinical targets that correlate with specific financial incentives is still an unanswered question. In addition, we did not specifically ask questions about clinical workflow, user interface characteristics, or information content; issues that our previous work indicated were important user acceptance factors [22,23]. Finally, a possible next step is to correlate actual clinician acceptance rates for various alert types with their stated actions (Note: A soon to be implemented version of our clinical information system software will enable us to track these events at the individual clinician level.)

## Conclusion

Based on the results of this survey we believe that patient and environmental characteristics are among the most important factors affecting the stated acceptance of various clinical decision support suggestions. There was no indication that any of the commonly thought of clinician characteristics such as age, years with the organization, or gender have much to do with the decision to accept or ignore various clinical decision support features. The fact that clinicians reported being behind schedule so often coupled with the finding that they stated they were much less likely to accept clinical decision support alerts when behind must be examined more closely. Finally, even though clinicians do not accept all of the clinical decision support that the system presents, overall clinicians indicate the benefits of these alerts still outweigh their costs.

## Competing interests

Dean F. Sittig is a member of Micromedex's Strategic Council. All the other authors declare that they have no competing interests.

## Authors' contributions

DFS, MAK, RHD, and HLC designed the survey. DFS managed the survey data collection process. DFS and AR analyzed and interpreted the data. DFS drafted the manuscript. All authors read and approved the final manuscript.

## Pre-publication history

The pre-publication history for this paper can be accessed here:



## Supplementary Material

Additional File 1 Clinical Decision Support Factors Questionnaire Click here for file
