# Supplementary material for: A survey of factors affecting clinician acceptance of clinical decision support
Source: BMC Med Inform Decis Mak. 2006 Feb 1;6:6. doi: 10.1186/1472-6947-6-6 (PMC1403751; doi:10.1186/1472-6947-6-6)
Supplement: Additional File 1 — Clinical Decision Support Factors Questionnaire [file 1472-6947-6-6-S1.doc]

Clinical decision support is clinical information that is either provided to you or accessible by you, from the EpicCare workstation. We consider enhanced displays such as flow sheets, health maintenance reminders, alternative medication suggestions, order sets or smart sets, alerts, and access to any internet-based information resources like the KPNW Clinical Library, as clinical decision support.

**For questions 1- 6 please enter a number in the blank, using the following scale:**

**1 2 3 4 5**

**| | | | |
 Never Rarely Some of the time Most of the time Always**

1. How often do you accept (i.e., do what the computer suggests) each of the following types of alerts:
2. Cost-related, medication suggestions _________ (1-5)
3. Safety-related, medication suggestions _______ (1-5)
4. Health maintenance reminders ______________ (1-5)
5. How often do you look up information on medications during a patient visit? ______ (1-5)
   What is your favorite resource: _________________________________________
6. When an alert appears while you are entering a medication order, how often do you feel:
7. Satisfied __________ (1-5)
8. Upset ____________ (1-5)
9. Relieved __________ (1-5)
10. Frustrated _________ (1-5)
11. Empowered _______ (1-5)
12. Mad __________ (1-5)
13. Helped ________ (1-5)
14. Grateful _______ (1-5)
15. Other feeling:
    ______________ ______ (1-5)
16. How would you “rate” the clinical decision support that is currently being offered within EpicCare:
17. It helps me take better care of my patients. ______ (1-5)
18. It’s worth the time it takes. ______ (1-5)
19. It reminds me of something I had forgotten about. ______ (1-5)
20. It _____________________________________________________________. ______ (1-5)
21. How often do you use the computer in the exam room during the patient visit to:
22. I have access to an exam room computer _______(1-5) (If NEVER, then skip the following questions!)
23. Look up patient information _______ (1-5)
24. Use KPNW Clinical Library or other reference information _______ (1-5)
25. Enter orders for the patient _______ (1-5)
26. Enter your progress / visit note _______ (1-5)
27. Show the patient a graph of his/her laboratory values, wt, blood pressure, or growth ______ (1-5)
28. How often are you:
29. Less than 20 minutes behind schedule _________ (1-5)
30. More than 20 minutes behind schedule ________ (1-5)
31. More than 40 minutes behind schedule ________ (1-5)
32. More than 60 minutes behind schedule _________ (1-5)
33. Of the patients you see with the following characteristics, are you MORE or LESS likely to accept health maintenance reminders:
34. Elderly patients (e.g., > 65 years old) ______MORE _______LESS ________ EQUALLY
35. Patients with many current medications (e.g., > 5) ______MORE _______LESS ________ EQUALLY
36. Patients with many chronic clinical conditions (e.g., >5) ______MORE _______LESS ________ EQUALLY
37. Patients presenting with an acute problem ______MORE _______LESS ________ EQUALLY
38. Are there other patient characteristics that make you ____MORE or ____LESS (check one) likely to accept an alert, If so, describe the characteristic(s) __________________________________________________.
39. I would be better off if I could _____INCREASE or _____DECREASE (check one) the number of alerts I see in my normal patient care routine.
40. When I am behind schedule, I am ____MORE or ____LESS or ____EQUALLY (check one) likely to accept an alert?
41. If I could turn OFF one specific alert, it would be? ___________________________________________ __________________________________________________________________________________________
    __________________________________________________________________________________________


    -------------------------- Fold Here ---------------------------------------------- Fold Here ------------------------------------

11. In an average ½ day clinical session, I get approximately _________ alerts or reminders that interrupt my workflow.

12. If you could change any aspect of clinical decision support within Epic, what would it be?

1. How old are you? _________years old
2. Are you ___MALE or ___FEMALE?
3. Years practicing at Kaiser Permanente: _________ years

Please

-------------------------- Fold Here ---------------------------------------------- Fold Here ------------------------------------

Staple and put in Inter-Departmental Mail.

Thanks.

Deliver to: Dean F. Sittig, Ph.D.

Center for Health Research

3800 N. Interstate Ave.

Portland, OR 97227
